# Supplementary material for: Ratiometric population sensing by a pump-probe signaling system in Bacillus subtilis
Source: Nat Commun. 2020 Mar 4;11:1176. doi: 10.1038/s41467-020-14840-w (PMC7055314; doi:10.1038/s41467-020-14840-w)
Supplement: Supplementary file 1 — Supplementary Information [file 41467_2020_14840_MOESM1_ESM.pdf]

Supplementary Information

Ratiometric population sensing by a pump-probe signaling system in *Bacillus subtilis*

Babel et al.

Supplementary Figures

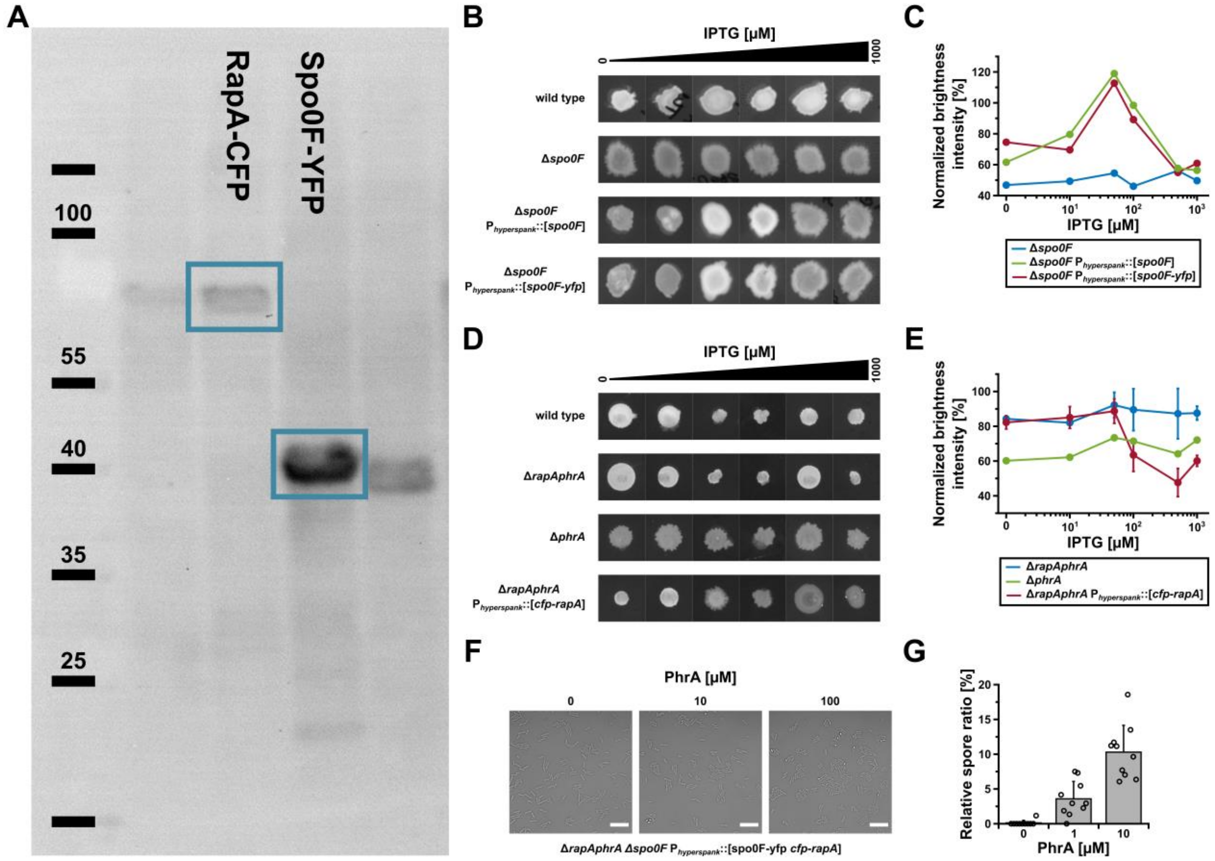

Supplementary Figure 1. Function of Spo0F-YFP and CFP-RapA fusions. (A) Western blot analysis of RapA-CFP and Spo0F-YFP. Protein sizes match those expected for the fusion proteins, as indicated by the green boxes. **(B-E)** Phenotypic test based on colonies grown for 3 days on DSM plates with IPTG to induce expression of the indicated proteins $P_{hyperspank}$. The opacity of a colony increases with activity in the sporulation phosphorelay (SPR) and thus, serves as a phenotypic read-out for protein function. **(B, C)** Spo0F-YFP complements a Δ*spo0F* background. **(B)** Cropped images of colonies grown on DSM plates with increasing IPTG-levels. **(C)** Grey-level colony intensity as a function of IPTG. Intensity values are expressed relative to a wt colony grown on the same plate. As YFP-Spo0F levels increase, colony opacity first increases and then decreases again and thus closely mirrors wild-type Spo0F, as observed previously [1]. **(D, E)** CFP-RapA inhibits the SPR. The DMS test was performed in a Δ*rapA phrA* background to avoid effects from PhrA inhibition of RapA. **(D)** Cropped images of colonies grown on DSM plates with increasing amounts of IPTG. **(E)** Measurements of colony opacity of CFP-RapA relative to wt as in (B). Induction of CFP-RapA decreases colony opacity. Error bars: mean ± SD from n_e = 3 plates. Data for Δ*rapA phrA* and Δ*phrA* is shown as a reference. **(F, G)** PhrA induces sporulation in the FRET reporter strain (BIB625) expressing Spo0F-YFP and RapA-CFP in a Δ*rapA phrA* Δ*spo0F* background. Sporulation was induced by the resuspension method by adding the indicated amount of PhrA to cells in RM with 10 µM IPTG. **(F)** Micrographs of bright-field images of unstimulated and PhrA-stimulated cells. PhrA facilitates sporulation, indicating that it counteracts CFP-RapA. **(G)** Sporulation frequency as a function of PhrA. Data points depict measurements from images with at least 80 objects. Values were normalized to wt-levels (BIB224). Error bars: mean ± SD from n_e = 10 images. Source data for all panels are provided as a Source Data file.

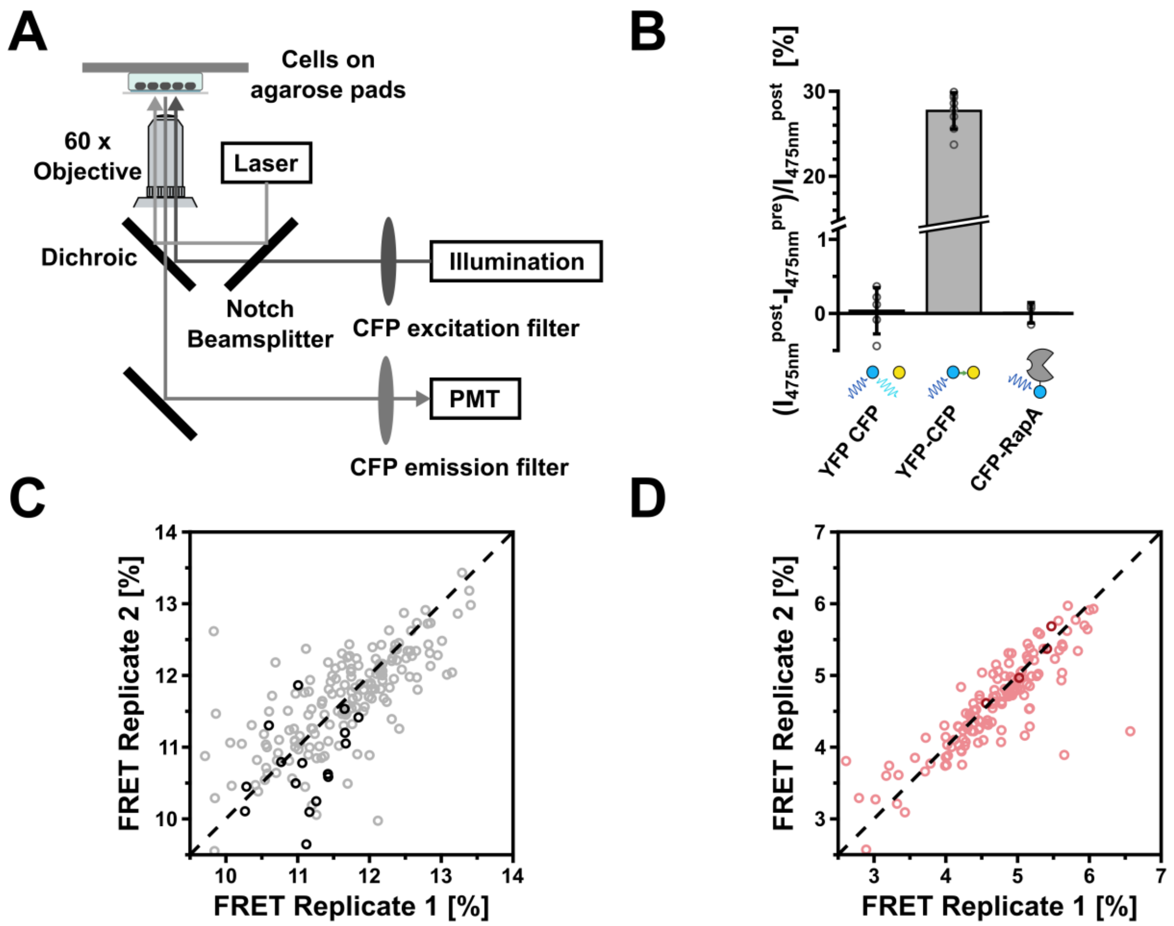

Supplementary Figure 2. Acceptor-photobleaching experiments quantify the FRET efficiency between CFP-RapA and YFP-Spo0F (A) Schematics of the set-up for acceptor photobleaching experiments. Changes in fluorescence in the donor channel (475 nm) upon bleaching of the acceptor with a 515 nm laser were measured on the average population level (i.e. hundreds of cells) using photomultiplier tubes (PMTs); see Methods for details. The data was validated by E-FRET imaging of individual cells (see Supplementary Fig. 8). **(B)** Bar-graph of FRET efficiencies for the indicated control strains. From left to right: a FRET negative control (BIB 138: YFP and CFP expressed separately, $n_e = 5$) and a FRET positive control (BIB134, expressing a YFP-CFP fusion protein, $n_e = 8$) and a donor-only control (BIB1902, $n_e = 3$). Data: mean ± SD from $n_e \geq 3$ experiments. Circles: Individual measurements from biological replicates. **(C, D)** Scatter plot of FRET efficiencies obtained from populations of (**C**) unstimulated and (**D**) stimulated (10 µM) reporter cells (BIB625) as determined over many independent experiments. The FRET efficiencies measured at two points on the same agarose pad are plotted against each other. The dashed line indicates the expected 1:1 correspondence of technical replicates. The variability in the FRET efficiency from technical replicates is much smaller than the overall variability from sample to sample, even for measurements taken more than 40 min apart (subset of black and dark red circles in **C** and **D**, respectively). Source data for all panels B-D are provided as a Source Data file.

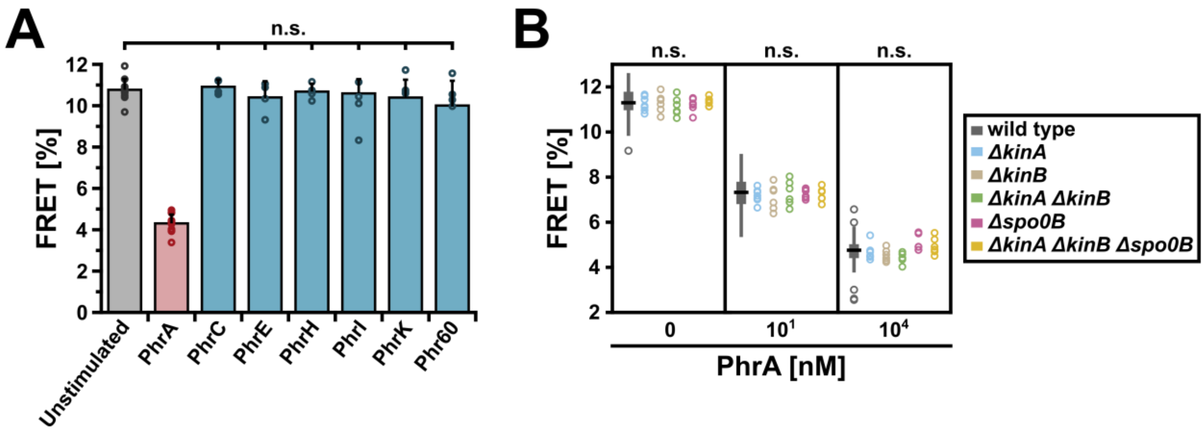

Supplementary Figure 3. Changes in FRET report specifically on effects of PhrA signaling via the RapA-Spo0F pathway. FRET reporter cells were stimulated with the indicated peptide as described in Methods. **(A)** FRET response of "WT" reporter cells (BIB625, *spo0F rapA phrA*) to stimulation with the cognate and non-cognate Phr signaling peptides (10 µM). Only PhrA decreases the FRET efficiency. Any other signaling peptides that act on Spo0F-targeting Raps do not affect FRET. Data: mean ± SD from $n_e \geq 4$ experiments. Statistical test: One-way ANOVA, $P = 0.52$ (n.s.). **(B)** Genetic perturbations to the sporulation phosphorelay have no effect on the FRET efficiency measured in unstimulated and PhrA-stimulated reporter cells that had received a non-saturating (10 nM) and a saturating stimulus (10 µM). The indicated genes *kinA, kinB* and/or *spo0B* were deleted from the "WT" reporter (BIB625). Differences in FRET efficiency between strains and WT are not significant. WT data is shown as a box-plot referencing to data shown in Fig. 2C with center line corresponding to the median, box-size 25% and 75% percentile, whiskers 99.3% and dots show outliers. Data: mean ± SD from $n_e = 6$ experiments. Statistical test: One-way ANOVA. 0 nM: $P = 0.98$ (n.s.), 10 nM: $P = 0.99$ (n.s.), 10^4 nM: $P = 0.32$ (n.s.). Legend: n.s.: $P > 0.05$, *: $P < 0.05$, **: $P < 0.01$, ***: $P < 0.001$. See Supplementary Data 1 for further statistical information on effect size and degrees of freedom. Source data for all panels are provided as a Source Data file.

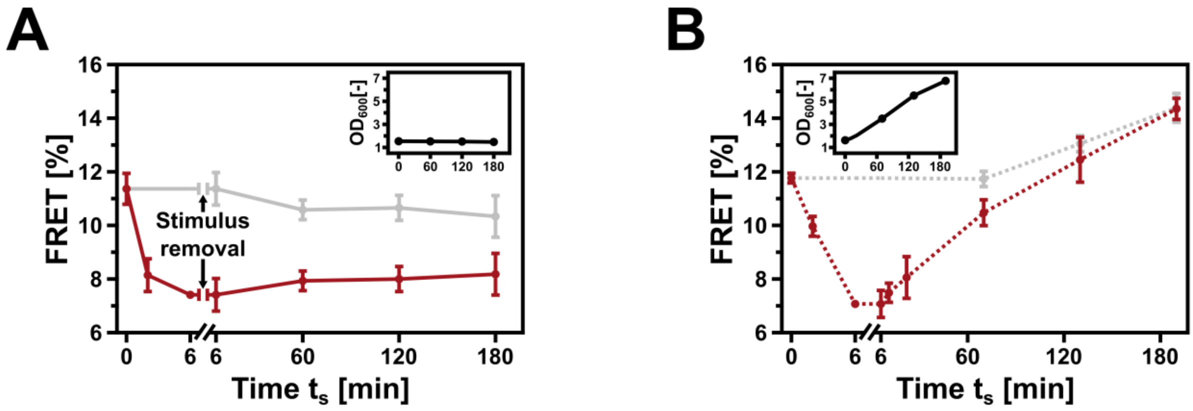

Supplementary Figure 4. Slow deactivation of stimulated cells. (A) FRET response upon stimulus removal in non-growing cells. Cells were exposed to a non-saturating stimulus (10 nM) for t_s = 5+1 min, resuspended in fresh medium and incubated at 37° C in a microtube on a thermoshaker. The curve of stimulated (red) and unstimulated controls (grey line) is shown. Data: mean ± SD from n_e = 4. Lines provide guidance to the eye. *Inset*: Corresponding OD_{600nm} curve. **(B)** FRET activation and recovery dynamics of growing cells in response to the addition of a 10 nM stimulus (red line). Note, FRET of unstimulated cells (grey line) rises after 1 hour of cultivation. Model fitting in Fig. 3C was based on data from the first hour only, during which FRET in unstimulated controls remained constant. *Inset*: Corresponding OD_{600nm} curve. Data: mean ± SD from n_e = 4. Lines provide guidance to the eye. Source data for all panels are provided as a Source Data file.

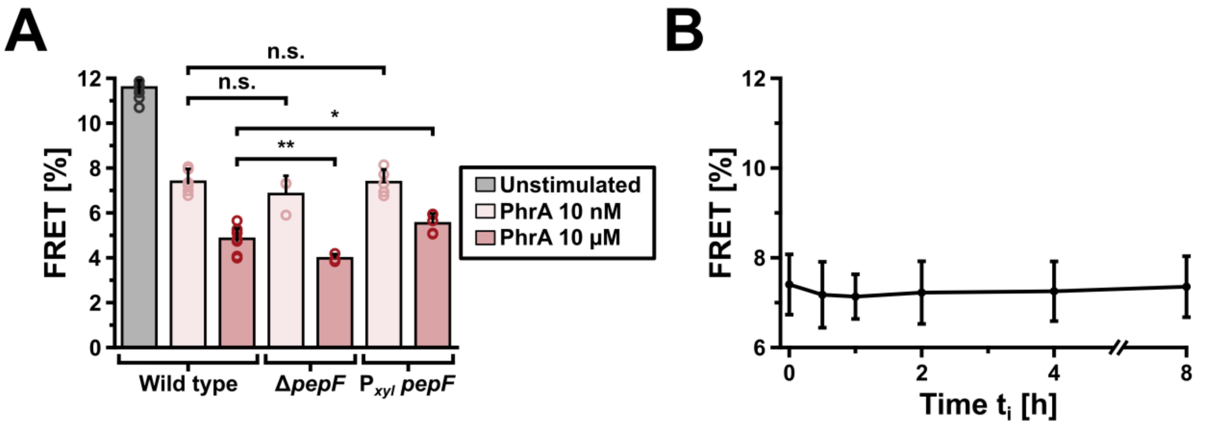

Supplementary Figure 5. Slow intra-and extracellular degradation of PhrA. (A) Effect of the cytoplasmic peptidase PepF on FRET. Bar plot of FRET values obtained (following stimulation with 10 nM and 10 µM PhrA for t_s =5+1 min) from wild-type reporter cells (WT, BIB625), and reporter cells lacking (*ΔpepF*, BIB1719) or overexpressing the peptidase PepF from a xylose-inducible promoter (P_{xyl} *pepF*, BIB1612). Data: mean ± SD from n_e = 3 for BIB1719 and n_e = 5 for BIB1612. While there is no significant difference when cells were stimulated with 10 nM, a 10 µM stimulus results in a small but significant decrease in FRET for Δ*pepF* and an increase in FRET for P_{xyl} *pepF*, respectively. Statistical test: Unpaired T-test. 10 nM Δ*pepF: P* = 0.37 (n.s.), 10 nM P_{xyl} *pepF: P* = 0.96 (n.s.), 10 µM Δ*pepF: P* = 0.004 (**), 10 µM P_{xyl} *pepF: P* = 0.03 (*). Legend: n.s.: $P > 0.05$, *: $P < 0.05$, **: $P < 0.01$, ***: $P < 0.001$. See Supplementary Data 1 for further statistical information on effect size and degrees of freedom. **(B)** PhrA is stable in cell-free supernatants. PhrA (10 nM) was added to cell-free supernatant and kept at room temperature for the specified amount of time t_i. The supernatants were then analyzed at the indicated times with the help of the bioassay described in Methods. FRET should increase over time if the signal were subject to degradation in the supernatant. However, FRET values remained unchanged at ~7 % for at least 8 h (black line). Data: mean ± SD from n_e = 3 experiments. Source data for all panels are provided as a Source Data file.

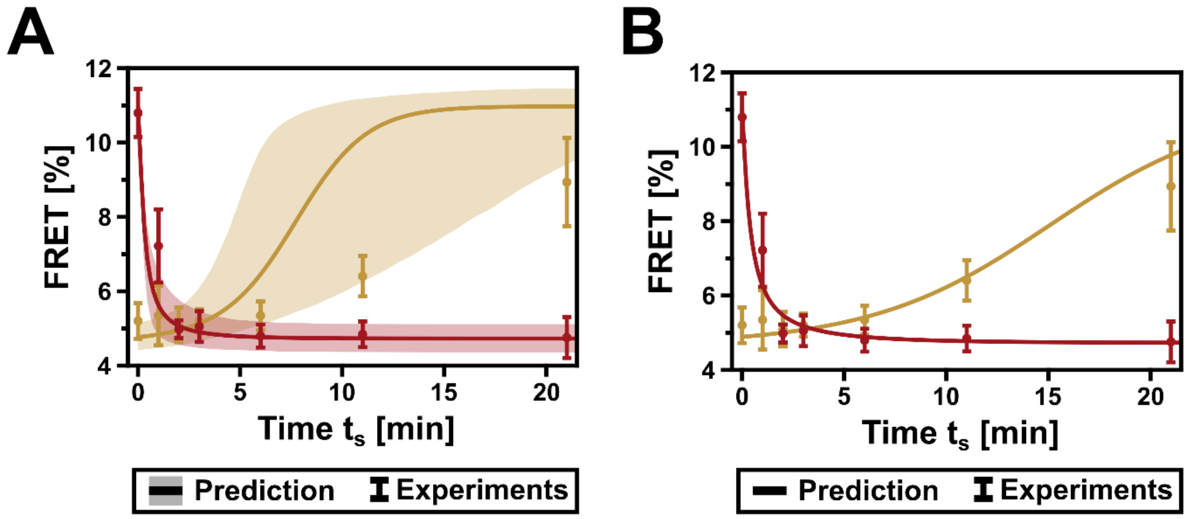

Supplementary Figure 6. Model prediction and refit of the response data to stimulation with 100 nM PhrA. (A) Model-based predictions with 95% confidence intervals (lines and shaded area) using the parameters in Table 1. The response dynamics to stimulation (red) is shown, together with the depletion of PhrA from the supernatants as measured by the bioassay (yellow). Experimental data: mean ± SD from $n_e = 7$. The intracellular response dynamics shows very good agreement with the prediction. However, the bioassay data falls close to the edge of the 95% confidence interval of the model prediction. **(B)** Results from re-fitting after inclusion of the 100 nM data set. The lines depict the best fit. The parameter estimates change by less than 25% compared to the values in Table 1, except for the maximal uptake rate v_{max} (-29%) and the effective K_M of signal import (+49%). However, the re-estimated parameters fit the 10 nM and 30 nM data less well (not shown). Thus, at higher PhrA concentrations, additional effects that are not captured by the simple pump-probe model may contribute to the extracellular signal dynamics. Source data for all panels are provided as a Source Data file.

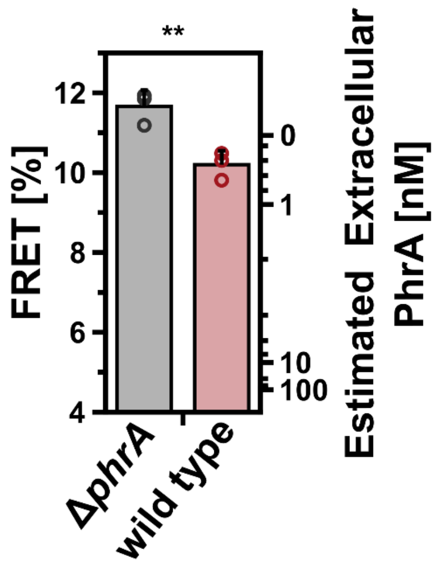

Supplementary Figure 7. The conditioned S7 supernatant from WT populations contains low levels of PhrA (sub-nM). Bar-graph of FRET efficiencies obtained from supernatant analysis with the help of a bioassay. The supernatants were obtained from WT cells (BIB224) grown in S7 media for 5 h under the conditions employed for the reporter cells (BIB625, relevant genotype Δ*phrA*). Supernatants were analyzed by the FRET reporter with the help of a sensitized bioassay as described in Material and Methods. Circles indicate measurements from biological replicates. The y-axis on the right converts FRET efficiencies into extracellular PhrA concentrations, as calculated on the basis of the pump-probe model. Data are presented as mean ± SD (n_e = 3). Unpaired T-test: P = 0.0099 (**). See Supplementary Data 1 for further statistical information on effect size and degrees of freedom. Source data are provided as a Source Data file.

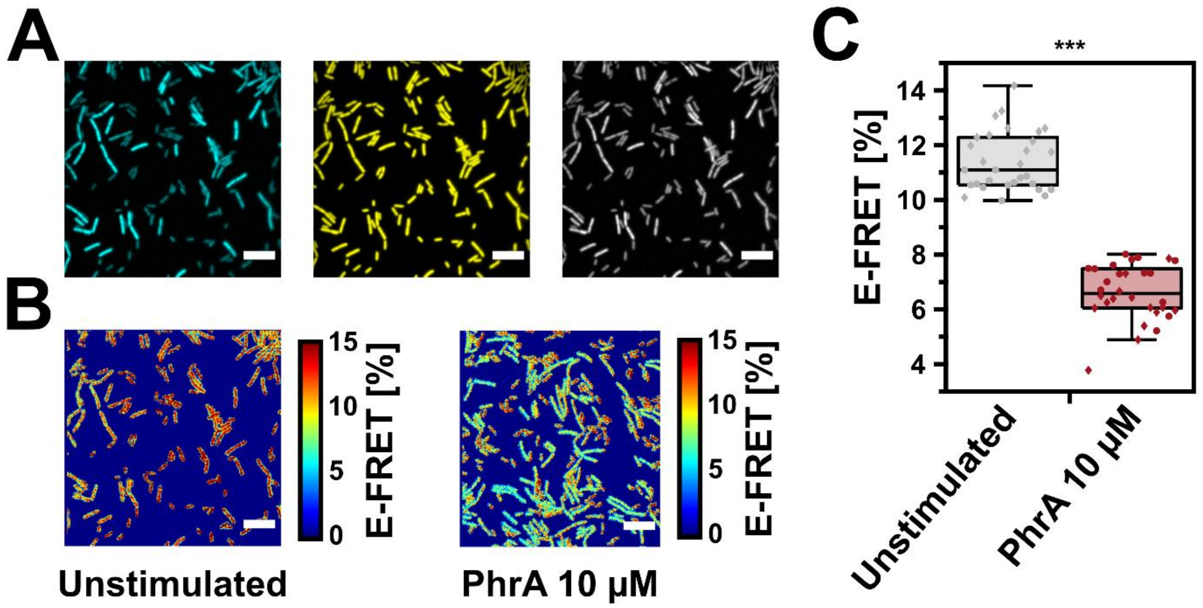

Supplementary Figure 8. Results of E-FRET imaging of unstimulated and PhrA-stimulated reporter cells. **(A)** CFP, YFP and FRET images of unstimulated reporter cells (BIB625). Scale bar: 10 µm. **(B)** E-FRET image of unstimulated (left) and PhrA-stimulated cells (right). Scale bar: 10 µm. See Methods for details. **(C)** Box plot of E-FRET data for unstimulated and stimulated populations. Each dot represents the average E-FRET from all cells on a single image as depicted in **B**. The data includes two biological replicates containing at least 15 images each. Center line: median, box-size: 25% and 75% percentile, whiskers: 99.3% and dots show outliers. Unpaired T-test: $P = 6*10^{-25}$ (***). See Supplementary Data 1 for further statistical information on effect size and degrees of freedom. Source data are provided as a Source Data file.

Supplementary Tables

Supplementary Table 1. Plasmids used in this study.

bla: ampicillin; *kanR*: kanamycin; *erm*: erythromycin; *specR*: spectinomycin; *cmR*: chloramphenicol

Plasmid	Description	Primers used for cloning	Reference or source
**pDR200**	*ori(F1) bla kanR ori(pUC) lacZ::eCFP*		David Rudner [2]
**AEC253**	*ori(pUC18) bla sacA:: spoIIA-yfp (venus; E. coli optimized) cmR*		Avigdor Eldar [3]
**pDR111**	*bla ori (ColE1) amyE:: specR rrnB T2 Phyperspank lacI*		David Rudner
**EIB151**	*bla ori (ColE1) amyE:: specR rrnB T2 Phyperspank yfp-cfp lacI*	yfp N-term for, yfp N-term rev, cfp C-term for, cfp C-term rev	This study
**EIB152**	*bla ori (ColE1) amyE:: specR rrnB T2 Phyperspank yfp cfp lacI*	yfp N-term for, yfp C-term rev, cfp N-term for, cfp C-term rev	This study
**EIB77**	*bla ori (ColE1) amyE:: specR rrnB T2 Phyperspank cfp-rapA lacI*	cfp N-term for, cfp N-term rev rapA N-term for, rapA N-term rev	This study
**EIB283**	*bla ori (ColE1) amyE:: specR rrnB T2 Phyperspank spo0F-yfp lacI*	yfp C-term for, yfp C-term rev spo0F C-term fwd, spo0F C-term rev	This study
**EIB284**	*bla ori (ColE1) amyE:: specR rrnB T2* $P_{hyperspank}$ *spo0F-yfp cfp-rapA lacI*		This study
**pDR111-LIC**	*bla ori (ColE1) amyE:: specR rrnB T2 Phyperspank LIC-site lacI*		Lorenz Adlung
**EIB139**	*bla ori (ColE1) amyE:: specR rrnB T2 Phyperspank spo0F lacI*	spo0F for spo0F rev	This study
**pAX01**	*bla ori (ColE1) lacA:: ermR* P_{xyl} *xylR*		[4]
**EIB544**	*bla ori (ColE1) lacA:: ermR* P_{xyl} *pepF xylR*	pepF for, pepF rev	This study

Plasmid	Description	Primer used for cloning	Reference or source
**pMAD**	*erm, ori(pE194-Ts), PclpB-bgaB, ori(pBR322), bla*		[5]
**EIB185**	*erm, ori(pE194-Ts), PclpB-bgaB, ori(pBR322), bla, up- and down-fragments of rapAphrA*	rapAphrA up-frag for rapAphrA up-frag rev rapAphrA down-frag for rapAphrA down-frag rev	This study
**EIB184**	*erm, ori(pE194-Ts), PclpB-bgaB, ori(pBR322), bla, up- and down-fragments of* phrA	phrA up-frag for phrA up-frag rev phrA down frag for phrA down frag rev	This study
**EIB213**	*erm, ori(pE194-Ts), PclpB-bgaB, ori(pBR322), bla, up- and down-fragments of* spo0B	spo0B up-frag for spo0B up-frag rev spo0B down-frag for spo0B down-frag rev	This study
**EIB216**	*erm, ori(pE194-Ts), PclpB-bgaB, ori(pBR322), bla, up- and down-fragments of kinA*	kinA up-frag for kinA up-frag rev kinA down-frag for kinA down-frag rev	This study
**EIB217**	*erm, ori(pE194-Ts), PclpB-bgaB, ori(pBR322), bla, up- and down-fragments of kinB*	kinB up-frag for kinB up-frag rev kinB down-frag for kinB down-frag rev	This study

**EIB221**	*erm, ori(pE194-Ts), PclpB-bgaB, ori(pBR322), bla, up- and down-fragments of spo0F*	spo0F up-frag for<br>spo0F up-frag rev<br>spo0F down-frag for<br>spo0F down-frag rev	This study
**EIB527**	*erm, ori(pE194-Ts), PclpB-bgaB, ori(pBR322), bla, up- and down-fragments of oppA*	oppA up-frag for<br>oppA up-frag rev<br>oppA down-frag for<br>oppA down-frag rev	This study
**EIB545**	*erm, ori(pE194-Ts), PclpB-bgaB, ori(pBR322), bla, up- and down-fragments of pepF*	pepF up-frag for<br>pepF up-frag rev<br>pepF down-frag for<br>pepF down-frag rev	This study

Supplementary Table 2. Oligonucleotides used in this study.
Restriction sites are in red, linker sequences are in bold, ribosome-binding sites in blue, LIC sites underlined, homologous sequences are in lower-case

Primers for construction of YFP and CFP fusion proteins	
**yfp c-term for**	**GGATCCGGAGGTGGAGTG**agcaaaggtgaagaactgttc
**yfp c-term rev**	TATATAGCTAGCgtaatccgcccgacggtatcatatg
**spo0F c-term for**	TATATAGTCGACaaatcataatattggggtgtaaaa
**spo0F c-term rev**	**CACTCCACCTCCGGATCC**gttagacttcaggggcagata
**cfp n-term for**	TCTAGAGCTAGCctaggctcgagaagcttacataaggaggaactac
**cfp n-term rev**	**CACTCCACCTCCGGATCC**cttataaagttcgtccatgc
**rapA n-term for**	**GGATCCGGAGGTGGAGTG**ttgaggatgaagcagacgat
**rapA n-term rev**	TATATAGCATGCgaccgcaacgagcaacaaacctgacatc

Primers for construction of YFP and CFP fusion proteins	
**yfp n-term for**	TATATAGTCGACcaaggaggaatgagggatcc
**yfp n-term rev**	**CACTCCACCTCCGGATCC**tttatacagttcgtccatacc
**cfp c-term for**	**GGATCCGGAGGTGGAGTG**gtttcaaaaggcgaagaact
**cfp c-term rev**	TATATAGCATGCgatccttacttataaagttcgtccatgccaagtgt

Primers for construction of PepF and Spo0F expression	
**pepF for**	TAACAACTAGTTAGGAGGAGAGGAAgtgaaaggcacaaaaggaaag
**pepF rev**	TGATAGGATCCttaagactgcttgactttcatcagc
**spo0F for**	AAGGAGGAAGCCACT atgatgaatgaaaaaattttaatc
**spo0F rev**	GACACGCACGCACT ttgtcagttagacttcagg

Primers for construction of clean deletion plasmids	
**rapAphrA up-frag for**	CCAGTCGACtatcaaacatatgacatcccttac
**rapAphrA up-frag rev**	GGTCTTTTTTTATGCATCATatcctcaattaatcccccctttg
**rapAphrA down-frag for**	Atgatgcataaaaaaagacccttag
**rapAphrA down-frag rev**	CATAGATCTtcggttaagcggcagaccttgaatc
**phrA up for**	CCAGTCGACagcagaagatgtttgtcagtgc
**phrA up rev**	GTCTTTTTTTATGCATCATttagatttcatataaacaatctcc
**phrA down for**	atgatgcataaaaaaagacccttag
**phrA down rev**	TTACCATGGttctcacccgatcgatgccccagc
**spo0F up-frag for**	CCAGTCGACctgtcgaagctgcaaatcagaag
**spo0F up-frag rev**	GTT*CG*AGACTTCAGGGGCAGAcatcattttacaccccaatattatg
**spo0F down-frag for**	Tctgcccctgaagtct*cg*aactgac
**spo0F down-frag rev**	GGGAGATCTttccgatctcttccatttctttg
**oppA up-frag for**	TACGCGTCGACgatataggcgacacactgcatc

**oppA up-frag rev**	CATCTGAATGAAGTCAGTTGcatgtgttttctgcccatattgc
**oppA down-frag for**	CAACTGACTTCATTCAGATGctcaaggtatatggggagaaaag
**oppA down-frag rev**	ATGGAAGATCTctaggctcctttctttgctttg
**pepF up-frag for**	TGATAGGATCCtggcagtttatcaatgcaagtcc
**pepF up-frag rev**	GCTTACTTTTagcctgcccaccacctttg
**pepF down-frag for**	TGGGCAGGCTaaaagtaagcctgtgcggaaatg
**pepF down-frag rev**	ATGGAAGATCTtgagatgcttggcgacgagc
**kinA up-frag for**	CCCGTCGACcgatctactgaaaatgagaaac
**kinA up-frag rev**	tccacagaatccctcctttgc
**kinA down-frag for**	GCAAAGGAGGGATTCTGTGGAggcacagcgtttaaaatttgcatttcc
**kinA down-frag rev**	TATAGATCTcggggccaagcctgtcattgttg
**kinB up-frag for**	TTTGTCGACatgcaatcctcataacccttctgg
**kinB up-frag rev**	CAATCGATTTGCTAGTGAGGttccattcgtgtgaaatcctttc
**kinB down-frag for**	CCTCACTAGCAAATCGATTGGAAC
**kinB down-frag rev**	CATAGATCTacaattattacgtggggaaacatgg
**spo0B up-frag for**	CCAGTCGACgagccgcttcttgatataggagag
**spo0B up-frag rev**	CTAGTCCACACCCAATTTCAActtcattttcgcactcccaatc
**spo0B down-frag for**	Ttgaaattgggtgtggactagcggag
**spo0B down-frag rev**	TTACCATGGtcatatggatcacggccttctaag

Primers for verification of strains	
**ΔrapAphrA locus for**	cgaacaagtggaaagacgtaag
**ΔrapAphrA locus rev**	atggggtttattgatggattac
**Δspo0F locus for**	gtttgtcattatcatttctctg
**Δspo0F locus for**	agaggataacaatgccaaactg
**ΔoppA locus for**	ggatcattcagagacatgatacgtg
**ΔoppA locus rev**	gcatctttccagagggaaagg
**ΔpepF locus for**	atggcaagaatcaaggagcatatg
**ΔpepF locus rev**	atggatgtttctgttaatccgac
**ΔkinA locus for**	gatgaaatttccattgtttctgagc
**ΔkinA locus rev**	gggtgtaaataatgggtaccttag
**ΔkinB locus for**	gctgtactcacttggccagacc
**ΔkinB locus rev**	ccgaaacgatcagcaagcatg
**Δspo0B locus for**	ctgtaagcatccgattattatag
**Δspo0B locus rev**	acctgacctatcctccaaatg
**amyE for**	ctggtcggagattgggatgatag
**amyE rev**	aatttccatgttgcgtaagtcag
**lacA for**	ttccattccgatggatttagac
**lacA rev**	acgtgcttctccttctcaatg
**ampR for**	atgagtattcaacatttccgtgtc
**ampR rev**	ttaccaatgcttaatcagtgagg
**dhl for**	cgtgcagatgcgaatac
**dhl rev**	cgccatgacttcactaac

Supplementary Table 3. *Bacillus subtilis* strains used in this study.

Strains	Genotype	Reference or source
**W168**	*trpC2 r+Mm+M*	Oskar Kuipers (1A700)
**BIB 134**	W168 *amyE:: specR rrnB T2 $P_{hyperspank}$ yfp-cfp lacI*	This study
**BIB 138**	W168 *amyE:: specR rrnB T2 $P_{hyperspank}$ yfp cfp lacI*	This study
**BIB 38**	W168 *spo0F::tet*	Daniel Lopez
**BIB 175**	W168 *spo0F::tet amyE:: specR rrnB T2 $P_{hyperspank}$ spo0F-yfp lacI*	This study
**BIB 177**	W168 *spo0F::tet amyE:: specR rrnB T2 $P_{hyperspank}$ spo0F lacI*	This study
**BIB 275**	W168 *ΔphrA*	This study
**BIB 279**	W168 *ΔrapAΔphrA*	This study
**BIB 370**	W168 *ΔrapAΔphrA amyE:: specR rrnB T2 $P_{hyperspank}$ cfp-rapA lacI*	This study
**BIB 415**	W168 *ΔrapAΔphrAΔspo0F*	This study
**BIB 623**	W168 *ΔrapAΔphrAΔspo0F amyE:: specR rrnB T2 $P_{hyperspank}$ spo0F-yfp lacI*	This study
**BIB 625/914**	W168 *ΔrapAΔphrAΔspo0F amyE:: specR rrnB T2 $P_{hyperspank}$ spo0F-yfp cfp-rapA lacI*	This study
**BIB 1554**	W168 *ΔrapAΔphrAΔspo0F ΔkinA amyE:: specR rrnB T2 $P_{hyperspank}$ spo0F-yfp cfp-rapA lacI*	This study
**BIB 1563**	W168 *ΔrapAΔphrAΔspo0F amyE:: specR rrnB T2 $P_{hyperspank}$ spo0F-yfp cfp-rapA lacI ΔoppA*	This study
**BIB 1612**	W168 *ΔrapAΔphrAΔspo0F amyE:: specR rrnB T2 $P_{hyperspank}$ spo0F-yfp cfp-rapA lacI lacA:: ermR P_{xyl} pepF xylR*	This study
**BIB 1719**	W168 *ΔrapAΔphrAΔspo0F ΔpepF amyE:: specR rrnB T2 $P_{hyperspank}$ spo0F-yfp cfp-rapA lacI*	This study
**BIB 1615**	W168 *ΔrapAΔphrAΔspo0F ΔkinB amyE:: specR rrnB T2 $P_{hyperspank}$ spo0F-yfp cfp-rapA lacI*	This study
**BIB 1616**	W168 *ΔrapAΔphrAΔspo0F ΔkinA ΔkinB amyE:: specR rrnB T2 $P_{hyperspank}$ spo0F-yfp cfp-rapA lacI*	This study
**BIB 1902**	W168 *ΔrapAΔphrAΔspo0F amyE:: specR rrnB T2 $P_{hyperspank}$ cfp-rapA lacI*	This study
**BIB 1910**	W168 *ΔrapAΔphrAΔspo0F amyE:: specR rrnB T2 $P_{hyperspank}$ lacI*	This study
**BIB 1993**	W168 *ΔrapAΔphrAΔspo0F Δspo0B amyE:: specR rrnB T2 $P_{hyperspank}$ spo0F-yfp cfp-rapA lacI*	This study
**BIB 1995**	W168 *ΔrapAΔphrAΔspo0F ΔkinA ΔkinB Δspo0B amyE:: specR rrnB T2 $P_{hyperspank}$ spo0F-yfp cfp-rapA lacI*	This study

Supplementary References

1. Sen, S., Garcia-Ojalvo, J. & Elowitz, M. B. Dynamical consequences of bandpass feedback loops in a bacterial phosphorelay. *PLoS One* **6**, e25102 (2011).
2. Doan, T., Marquis, K. A. & Rudner, D. Z. Subcellular localization of a sporulation membrane protein is achieved through a network of interactions along and across the septum. *Mol. Microbiol.* **55**, 1767–81 (2005).
3. Nagai, T. *et al.* A variant of yellow fluorescent protein with fast and efficient maturation for cell-biological applications. *Nat. Biotechnol.* **20**, 87–90 (2002).
4. B., H. *et al.* Development of a New Integration Site within the Bacillus subtilis Chromosome and Construction of Compatible Expression Casettes. *J. Bacteriol.* **183**, 2696–2699 (2001).

5. Arnaud, M., Chastanet, A. & De, M. New Vector for Efficient Allelic Replacement in Naturally Gram-Positive Bacteria. *Appl. Enviromental Microbiol.* **70**, 6887–6891 (2004).
