# Supplementary material for: Ratiometric population sensing by a pump-probe signaling system in Bacillus subtilis
Source: Nat Commun. 2020 Mar 4;11:1176. doi: 10.1038/s41467-020-14840-w (PMC7055314; doi:10.1038/s41467-020-14840-w)
Supplement: Supplementary file 3 — Description of Additional Supplementary Files [file 41467_2020_14840_MOESM3_ESM.pdf]

Description of Additional Supplementary Files

File Name: Supplementary Data 1

Description: Statistical information on effect sizes and degrees of freedom for Fig. 3C, Fig. 5C, Supplementary Fig. 3A & B, Supplementary Fig. 5A, Supplementary Fig. 7 and Supplementary Fig. 8C.
